# Oxygen-loaded microbubble-mediated sonoperfusion and oxygenation for neuroprotection after ischemic stroke reperfusion

**DOI:** 10.1186/s40824-023-00400-y

**Published:** 2023-07-06

**Authors:** Yi-Ju Ho, Hsiang-Lung Cheng, Lun-De Liao, Yu-Chun Lin, Hong-Chieh Tsai, Chih-Kuang Yeh

**Affiliations:** 1 Department of Biological Science and Technology, National Yang Ming Chiao Tung University, Hsinchu, Taiwan; 2 Center for Intelligent Drug Systems and Smart Bio-devices (IDS2B), National Yang Ming Chiao Tung University, Hsinchu, Taiwan; 3 Department of Biomedical Engineering and Environmental Sciences, National Tsing Hua University, No. 101, Section 2, Kuang-Fu Road, Hsinchu, 30013 Taiwan; 4 Institute of Biomedical Engineering and Nanomedicine, National Health Research Institutes, Zhunan, Taiwan; 5 Department of Medical Science, National Tsing Hua University, Hsinchu, Taiwan; 6 Institute of Molecular Medicine, National Tsing Hua University, Hsinchu, Taiwan; 7 Department of Neurosurgery, Linkou Chang Gung Memorial Hospital, No.5Fuxing St.Guishan Dist., Taoyuan City, 333 Taiwan; 8 School of Traditional Chinese Medicine, Chang Gung University, Taoyuan, Taiwan

**Keywords:** Ultrasound, Thrombolysis, Perfusion, Ischemia–reperfusion injury, Inflammatory responses, Mechanical effect

## Abstract

**Background:**

Ischemic stroke-reperfusion (S/R) injury is a crucial issue in the protection of brain function after thrombolysis. The vasodilation induced by ultrasound (US)-stimulated microbubble cavitation has been applied to reduce S/R injury through sonoperfusion. The present study uses oxygen-loaded microbubbles (OMBs) with US stimulation to provide sonoperfusion and local oxygen therapy for the reduction of brain infarct size and neuroprotection after S/R.

**Methods:**

The murine S/R model was established by photodynamic thrombosis and thrombolysis at the remote branch of the anterior cerebral artery. In vivo blood flow, partial oxygen pressure (pO_2_), and brain infarct staining were examined to analyze the validity of the animal model and OMB treatment results. The animal behaviors and measurement of the brain infarct area were used to evaluate long-term recovery of brain function.

**Results:**

The percentage of blood flow was 45 ± 3%, 70 ± 3%, and 86 ± 2% after 60 min stroke, 20 min reperfusion, and 10 min OMB treatment, respectively, demonstrating sonoperfusion, and the corresponding pO_2_ level was 60 ± 1%, 76 ± 2%, and 79 ± 4%, showing reoxygenation. After 14 days of treatment, a 87 ± 3% reduction in brain infarction and recovery of limb coordination were observed in S/R mice. The expression of NF-κB, HIF-1α, IL-1β, and MMP-9 was inhibited and that of eNOS, BDNF, Bcl2, and IL-10 was enhanced, indicating activation of anti-inflammatory and anti-apoptosis responses and neuroprotection. Our study demonstrated that OMB treatment combines the beneficial effects of sonoperfusion and local oxygen therapy to reduce brain infarction and activate neuroprotection to prevent S/R injury.

**Graphical Abstract:**

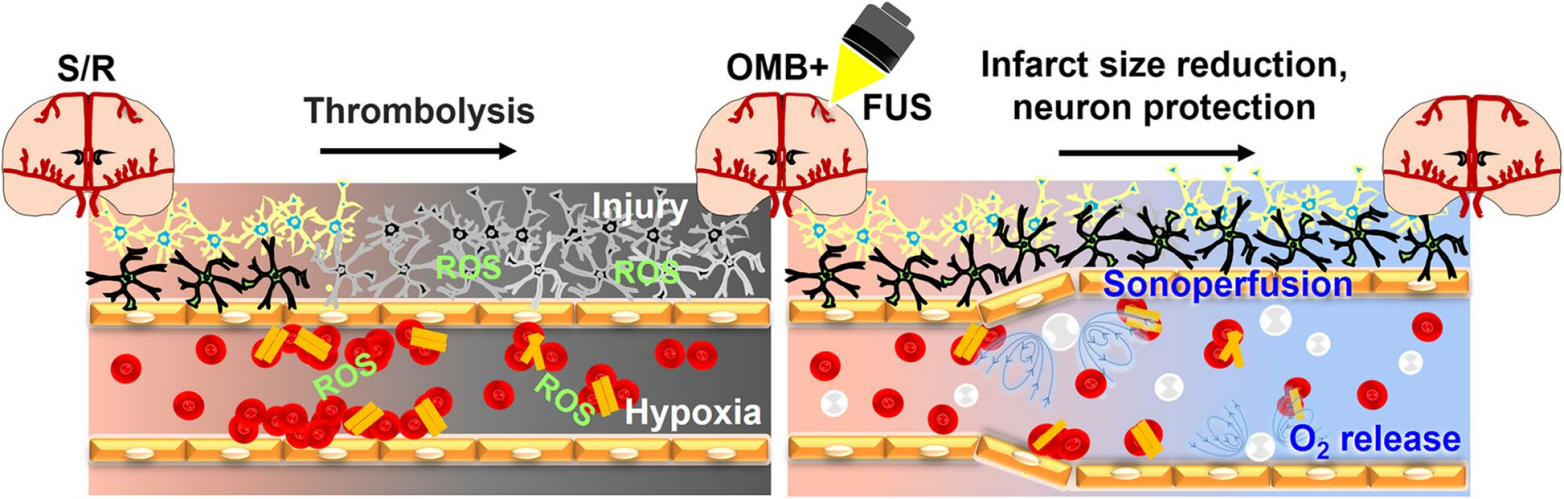

**Supplementary Information:**

The online version contains supplementary material available at 10.1186/s40824-023-00400-y.

## Background

Ischemic stroke is one of the leading causes of disability and mortality worldwide. Obstruction of blood flow delivery due to thrombus after ischemic stroke induces neuronal damage and dysfunction that result in limb incoordination, cognitive deterioration, and central nervous system diseases. Intravenous injection of tissue plasminogen activator (tPA) for thrombolysis has been approved by the United States Food and Drug Administration (FDA) for the treatment of acute ischemic stroke within 3 h of onset [1]. However, the reperfusion of blood flow after thrombolysis can further damage the ischemic vessels and penumbra, causing ischemic stroke-reperfusion (S/R) injury [2]. The induction process of S/R injury includes reactive hyperemia, hypoperfusion, and increased paracellular permeability, resulting in blood–brain barrier (BBB) disruption, nutritional deficiency, and neutrophil adhesion that increase inflammatory and oxidative stress [3]. Finally, the irreversible ischemic penumbra will enlarge the infarct size and aggravate neuron death, reducing the prognosis of ischemic stroke after thrombolysis.

Current thrombolytic therapy for ischemic stroke is usually combined with neuroprotective agents to protect ischemic neurons and prevent S/R injury by clearing free radicals and inhibiting inflammatory and apoptotic factors [4]. However, these neuroprotective agents showed potential in animal models but have failed in clinical trials [5]. The main reasons for failure of this strategy might be an insufficient treatment dose of neuroprotective agents within the ischemic brain, unfavorable long-term vessel recanalization, and inability to restore the microcirculation [5, 6]. Furthermore, establishing the balance of neuroprotective agents between an effective treatment dose and induction of systemic side effects (such as disruption of the central nervous system or gastrointestinal system or reversible altered mentation) should be considered [4]. Since the nutrient supply for neurons relies on the adjacent microcirculation, effective reperfusion within the microcirculation has been reported as a key factor to maintain neuronal function [7].

Ultrasound (US) has been widely applied to provide real-time medical imaging for clinical diagnosis. One commercial contrast agent for US imaging is microbubbles (MBs), which are designed on the basis of the high difference in acoustic impedance between soft tissue and gas [8]. Improved contrast imaging due to the presence of intravascular MBs provides further information about microvascular distribution and blood perfusion to evaluate vascular health, tumor progression, or cardiovascular diseases [9]. Upon US stimulation of intravascular MBs, the cavitation effects produced by MB oscillations generate additional mechanical forces on the vessel wall that can induce enhanced permeability for drug penetration, vasoconstriction for blood supply blockage, or vasodilation for enhanced blood perfusion [10–12]. US-stimulated MB cavitation has been proposed as a potential way to prevent ischemia–reperfusion injury in myocardial infarction, ischemic stroke, and peripheral arterial diseases via acousto-mechanical vasodilation, termed sonoperfusion [13–15]. Furthermore, MBs can also be used to carry drugs, genes, or specific gases for local delivery in the reperfusion regions to reduce injury and systemic side effects [16–18].

In the present study, oxygen-loaded MBs (OMBs) were used to examine the concept of local oxygen (O_2_) therapy triggered by US to prevent S/R injury. Since O_2_ plays an important role in cell metabolism through the generation of energy, additional compensation for O_2_ loss in the ischemic brain might be a promising way to maintain neuronal function. US-stimulated OMB treatment has been applied to enhance tumor oxygenation through local O_2_ release, which modified tumor microenvironment to prevent hypoxia, induce vascular normalization, and improve the efficacy of radiotherapy and sonodynamic therapy [19–21]. Our previous study has been proved that OMB treatment provided sonoperfusion and local O_2_ therapy to enhance effective reperfusion in the microcirculation and promote repair of injured cells through aerobic respiration in the murine hindlimb and cardiac ischemia–reperfusion models [22]. Therefore, we established a murine S/R model after tPA-induced thrombolysis to investigate the mechanisms and results of OMB treatment. In vivo tracking of blood flow and tissue O_2_ levels revealed the real-time OMB treatment conditions of a targeted vessel. Long-term follow-up of brain infarct size and animal behavior was performed to assess recovery of brain function. Finally, protein and mRNA expression was evaluated to determine the mechanisms of OMB treatment.

## Methods

### OMB preparation

The details and optimal fabrication of OMB were followed our previous study [21, 23]. The lipid shell of OMB contained 1,2 Distearoyl-sn-glycero-3-phosphorylcholine (DSPC) and 1,2-distearoyl-sn-glycero-3-phosphoethanolamine-N-[10-(trimethoxysilyl)undecanamide(polyethylene glycol)-2000] (DSPE-PEG2000; Avanti Lipids Polar, Alabaster, USA) with a weighted ratio of 10:4. The gas core contained perfluoropropane (C_3_F_8_) and O_2_ with a volume ratio of 7:5, which was regulated by syringes to exchange some of the C_3_F_8_ with O_2_ in a sealed vial. The OMBs provided both local O_2_ therapy and sonoperfusion. MBs loaded with pure C_3_F_8_ (CMBs) were used as control MBs for evaluation of sonoperfusion. The size distribution and concentration of OMBs and CMBs were detected using a particle size analyzer (Multisizer 3, Beckman Coulter, Fullerton, USA).

### Stability

The in vitro stability of OMBs was evaluated by contrast enhancement under US B-mode imaging since MBs function as a US contrast agent. A cylindrical hollow chamber (Ø = 5 mm) within a 2% cuboid agarose phantom was made to simulate the vascular lumen within tissues. The phantom was placed in a water tank maintained at a temperature of 37 °C. Diluted OMB emulsion (2 × 10^7^ MBs/mL, the same MB dose as for in vivo experiments) was placed in the cylindrical hollow chamber and a commercial US imaging system (Terason, Model 3000, Burlington, USA) with a 7-MHz linear array transducer was applied to record transverse sectional images of the chamber. The time-sequence US images were collected with 5 images at each time point (0 to 60 min) and then analyzed using MATLAB (MathWorks, Natick, USA) software. A region of interest (ROI; 2.5 × 2.5 mm^2^) was placed at the center of the cylindrical hollow on US images to quantify the increase in contrast intensity in the presence of OMBs.

### Destruction threshold

For local release of O_2_ from OMB disruption triggered by US, the destruction threshold of OMBs under various acoustic pressures was evaluated. A hollow pipe (Ø = 0.58 mm) within a 2% cuboid agarose phantom was connected to a PE50 tube (BD Corp., Franklin Lakes, USA) and a syringe pump (flow rate 1.6 μL/s) to generate a flowing condition that simulated OMB perfusion in the blood circulation. The concentration of OMB emulsion was 2 × 10^7^ MBs/mL. A 1-MHz focused US transducer (Spherical focus PFT, V302, Olympus, USA) was used to transmit 5000-cycle US pulses with different acoustic pressures (peak negative pressures of 0–600 kPa). The pulse repetition frequency (PRF) of 1 Hz was set according to the flow rate to confirm that OMBs were stimulated once within the US focal zone (diameter of 3 mm and length of 26 mm). A commercial US imaging system (Prodigy 128, L18.0VM, S-Sharp) with an 18-MHz linear array transducer was used to record transverse sectional images of the hollow pipe downstream of the US focal area. The intensity of contrast enhancement on US B-mode images was quantified using MATLAB software. A ROI (0.25 × 0.25 mm^2^) was placed at the center of the hollow pipe on US images to quantify the contrast intensity induced by the remaining OMBs after US stimulation.

### O_2_ release

The in vitro O_2_ release from OMB disruption was demonstrated by detecting the partial pressure of O_2_ (pO_2_) of the OMB emulsion. The experimental set up was the same as for the stability study. The concentration of CMB and OMB emulsion was 2 × 10^7^ MBs/mL. For real-time detection of the pO_2_ level, a fiberoptic probe was inserted into the cylindrical hollow chamber of the phantom. The pO_2_ levels were received by an OxyLite 2000 system (Oxford Optronics) at pre, during, and post 10-min US sonication (1 MHz, 5000-cycle, PRF 1 Hz, 300 kPa). In addition, OMB emulsions with different concentrations of MBs (1 × 10^7^, 2 × 10^7^, 4 × 10^7^ MBs/mL) were used to evaluate the amount of O_2_ release during US sonication and demonstrate the encapsulation of O_2_ within OMBs. The percentage of contrast intensity under the different acoustic pressures was calculated relative to the control group without US stimulation.

### SCD/ICD

The stable cavitation dose (SCD) and inertial cavitation dose (ICD) of OMBs were determined to evaluate the type of MB oscillation during US stimulation. The demarcation of acoustic pressures between SCD and ICD would allow release O_2_ from OMB disruption while avoiding damage to cells and vessels. The diluted OMB emulsion (2 × 10^7^ MBs/mL) was infused into a cellulose tube (Ø = 200 μm; Spectrum Labs, USA) that mimicked vessels with a flow rate of 0.375 mL/h. A 1-MHz focused US transducer was used to transmit therapeutic US pulses with different acoustic pressures (5000-cycle, PRF 1 Hz, 0–600 kPa). A 0.5-MHz or 5-MHz US receiving transducer (Spherical focus PFT, V301 and V307, Olympus, USA) was placed perpendicular to the 1-MHz focused US transducer. The US receiving transducers were used to detect the scattered signals from the oscillating OMBs during US stimulation. The detailed methods of signal analysis were described in our previous study [24].

### Murine ischemia-stroke reperfusion model

All animal experimental procedures were performed with approval from the Animal Experiment Committee at National Yang Ming Chiao Tung University (approval number: 111025A). 182 Mice (C57BL/6JNarl, male, 8–14 weeks old, 25–30 g) were purchased from the National Laboratory Animal Center (Taipei, Taiwan). Before the experiment, mice were intraperitoneally anesthetized with a mixture of Zoletil 50 (Virbac, Carros, France) and Rompun 2% (Bayer HealthCare, Leverkusen, Germany). The photodynamic dyes, drugs, and MBs were retro-orbitally administrated using a manual insulin needle. The body temperature of mice was maintained at 35–37 °C using a heating pad (THM100, Indus Instruments, Houston, USA).

**Fig. 1:**
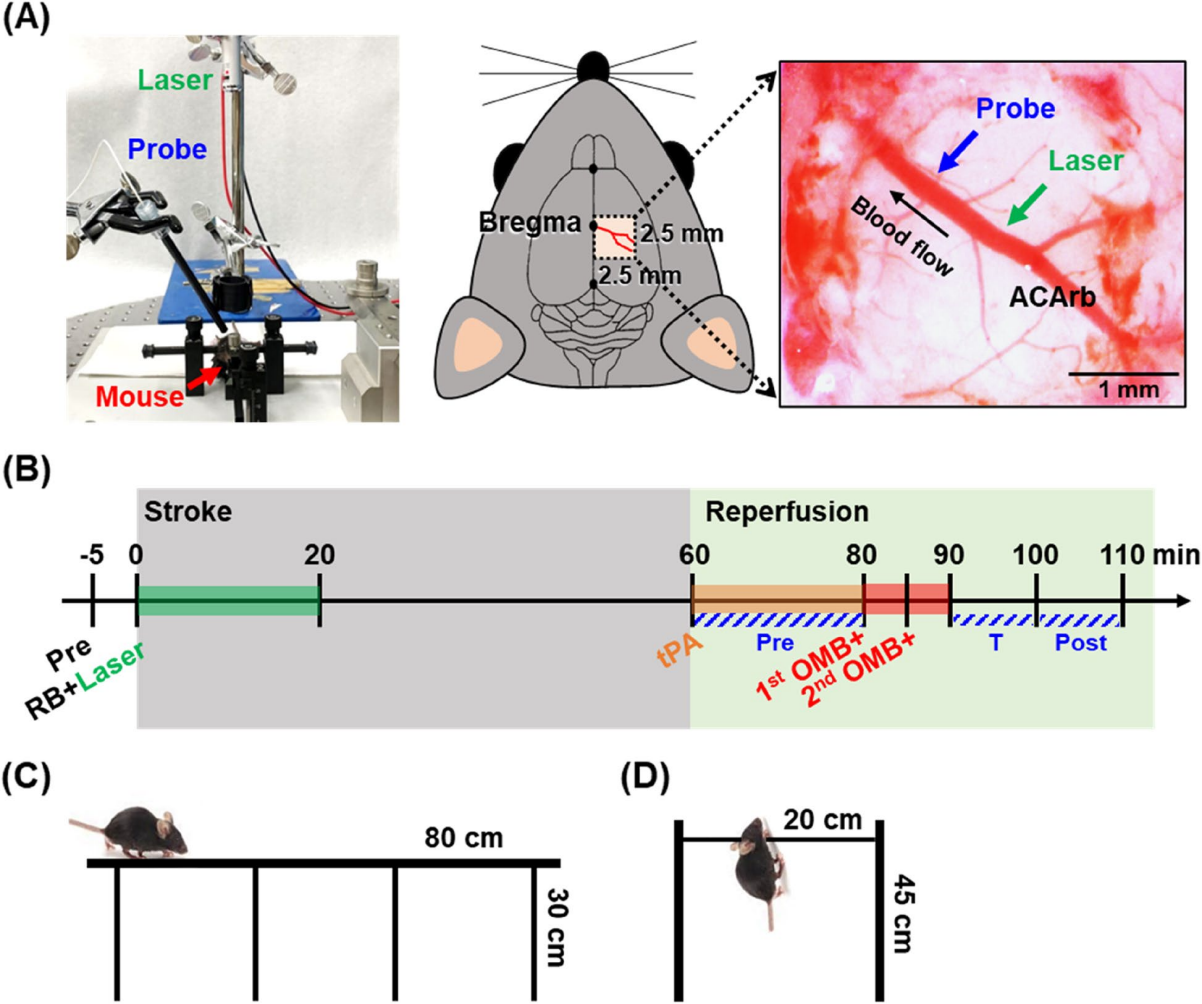
Methods for murine stroke/reperfusion (S/R) model establishment, treatment, and behavior assessments. **A** The experimental setup for establishment of the S/R model. The left photo shows the relative positions of the laser, fiberoptic probe, and mouse. The middle illustration indicates the location of a cerebral window (2.5 mm × 2.5 mm) located posterior and lateral to the bregma in the right cerebral hemispheres to uncover the anterior cerebral artery remote branch (ACArb). The right image reveals the relative locations of the focus of laser exposure and the detection point of the fiberoptic probe at ACArb. **B** Flow chart showing establishment of murine S/R model and OMB treatment. **C**, **D** Schematic illustration of **C** beam walking test and **D** hanging wire test for assessment of animal behavior

In this study, photodynamic thrombosis was used to generate a clot at the murine anterior cerebral artery remote branch (ACArb) to establish the ischemic stroke model. The experimental set up is illustrated in Fig. 1. Under anesthesia, a 2.5 mm × 2.5 mm piece of the skull located posterior and lateral to bregma (right brain) was removed to expose the ACArb (Fig. 1A). Mice were retro-orbitally injected with 50 μL of the photosensitizer rose bengal (RB, 10 or 20 mg/kg; Alfa Aesar, Bellingham, USA) for 30 s circulation and then exposed to a laser (532 nm, 0.5 mW) for 10, 15, or 20 min at the ACArb (~ 1.0 mm lateral to bregma; each *N* = 3–5) for the generation of clots and establishment of the ischemic stroke model. After 60 min of ischemia, mice were retro-orbitally injected with tPA (10 mg/kg; Actilyse, Boehringer Ingelheim, Ingelheim, Germany) for thrombolysis and reperfusion. Methods of tPA administration were (1) two 50 μL bolus injections with an interval of 10 min, designated 50 + 50 or (2) 10 μL bolus injection and 90 μL introduced by a syringe pump (KDS120, KD Scientific, New Hope, PA, USA) with a constant flow rate of 4.5 μL/min (total injection time: 20 min), designated 10 + 90 (each *N* = 3–5).

During establishment of the murine S/R model, a fiberoptic probe (NX-BF/OFT/E, Oxford Optronics, Oxford, UK) linked to OxyLite and OxyFlo 2000 systems (Oxford Optronix) was non-invasively placed downstream of ACArb (~ 0.5 mm lateral to bregma) for real-time detection of in vivo blood flow and pO_2_. A stereo microscope (SZ61, Olympus, Tokyo, Japan) was used to observe the process of thrombosis and thrombolysis at ACArb. After 24 h of reperfusion, the mice were sacrificed and perfused with 0.9% normal saline. The brains were removed, sliced into coronal sections with thickness of 2 mm, and stained with 2% 2,3,5-triphenyltetrazolium chloride (TTC; Sigma-Aldrich, St. Louis, USA) for 20 min at 37 °C. Finally, the brain sections were observed and images were recorded using a stereo microscope. Tissue images were analyzed using MATLAB software to quantify the infarct areas labeled white by TTC staining within the brain section. The percentage of the infarct area in brain tissue was calculated only for the sections that showed an infarct area, rather than for the entire brain. The blood flow, pO_2_, and infarct area were used to evaluate the optimal RB doses, laser exposure times, and tPA administration method for establishment of the murine S/R model.

### Biosafety and BBB opening

Our previous studies have demonstrated the in vitro cytotoxicity and in vivo safety of OMB administration, revealing non-significant toxicity of OMBs at the optimal treatment dose (2 × 10^7^ OMBs/mouse) [21–23]. Since MB cavitation could enhance vascular permeability, open the BBB, and even disrupt vessel walls, the biosafety of OMB cavitation should be considered. Our murine S/R model was used to evaluate the bioeffects after OMB treatment. A 1-MHz US transducer with acoustic pressure of 300 or 400 kPa was focused on the S/R site of ACArb. At 20 min after tPA injection, mice were retro-orbitally injected with different concentrations of OMB emulsion (0.5 × 10^7^, 1 × 10^7^, and 2 × 10^7^ OMBs in 50 μL; each *N* = 3) for 30 s circulation and then subjected to US sonication for 5 min to disrupt the OMB for local O_2_ therapy and sonoperfusion. The OMB treatment procedure included two rounds of OMB administration and US sonication; the second dose of OMBs was injected immediately after the first sonication. The total OMB treatment therefore consisted of 1 × 10^7^, 2 × 10^7^, or 4 × 10^7^ OMBs and 10 min US sonication. After OMB treatment, 50 μL of Evans blue dye (2.5 mL/kg) was retro-orbitally injected into the mice to visualize the BBB opening induced by OMB cavitation. After 3 h, mice were sacrificed and perfused with 0.9% normal saline. The brains were removed and sliced into coronal sections with a thickness of 2 mm. The brain sections were observed and images were recorded using a stereo microscope to evaluate BBB opening and brain hemorrhage. The bioeffects induced by different OMB doses and acoustic pressures were used to define the optimal OMB treatment protocol for subsequent experiments.

### OMB treatment

The treatment procedures of the murine S/R model involved the following steps (Fig. 1B): (1) 60 min stroke induction by photodynamic thrombosis; (2) 20 min of reperfusion induced by tPA injection; (3) 10 min OMB treatment including two rounds of OMB administration (1 × 10^7^ MBs/mouse/injection) and US sonication (1-MHz, 5000-cycle, PRF 1 Hz, 300 kPa, 10 min). The S/R mice were separated into six groups: S/R, CMB, OMB, S/R + , CMB + , and OMB + groups, where the plus ( +) symbol presents US sonication (each group *N* = 5). Real-time recording of blood flow and pO_2_ downstream of the ACArb was performed to evaluate establishment of the S/R model and OMB treatment effects. The mean percentages of blood flow and pO_2_ were determined during the periods of pre-treatment (pre), during OMB treatment (T), and post-treatment (post). After 24 h of reperfusion the mice were sacrificed for TTC staining to estimate the treatment efficacy based on changes in brain infarct size. It should be noted that the brain infarct size was calculated as the percentage of infarct areas within the whole brain, which was different from the method used in establishment of the S/R model.

### Post-treatment evaluation of animal behaviors and brain infarct size

Animal behaviors and brain infarct size were tracked over 14 days to evaluate the long-term recovery of brain function. Since the brain infarct was located at the primary and secondary motor areas, the beam walking test and hanging wire test were used to evaluate the motor coordination and muscle performance of S/R mice. Mice were separated into four groups (normal, S/R, CMB + , and OMB + groups, each *N* = 4–5) for evaluation of behaviors at 1, 3, 7, and 14 days after treatment. The beam walking test was performed five times to calculate the average walking times (Fig. 1C). In the hanging wire test, the average recorded time calculated from five repetitions was multiplied by the mouse weight to account for the influence of body size (Fig. 1D). In addition, the long-term effect of inflammatory responses on infarction was evaluated by determining the percentage of infarct areas within the whole brain at 1, 3, 7, and 14 days after treatment (each *N* = 3). The time point of maximum infarct size in the S/R group was chosen for further histological analysis and measurement of protein and mRNA expression.

### Histological qualitative assessment

At 3 days after OMB treatment the mice were sacrificed and perfused with 0.9% normal saline. The experimental groups included normal, S/R, CMB + , and OMB + groups (each *N* = 3). The brain tissue was removed for frozen sectioning at a thickness of 20 μm using a cryostat microtome (Leica CM1850 Cryostat, Leica Biosystems GmbH, Wetzlar, Germany). Hematoxylin and eosin staining (H&E) was used to observe brain tissue structure. Since brain infarction causes differentiation of microglia, apoptosis of neuron cells, and population with astrocytes, immunohistochemical assessments of staining for CD206 (anti-mannose receptor antibody, ab64693, Abcam, Cambridge, UK), CD11b (purified rat anti-CD11b, 550,282, BD Biosciences, Franklin Lakes, USA), NeuN (recombinant anti-NeuN antibody [EPR12763]-Neuronal Marker, ab177487, Abcam), and GFAP (anti-GFAP antibody, ab4674, Abcam) were performed to evaluate the distribution of anti-inflammatory M2 microglia, pro-inflammatory M1 microglia, mature neurons, and astrocytes, respectively, within the brain infarct areas.

### Enzyme-linked immunosorbent assay

At 3 days after OMB treatment the mouse brain was separated into the right S/R cerebral hemisphere and left contralateral cerebral hemisphere. The perfused cerebral hemispheres were collected and homogenized in 1 mL phosphate-buffered saline using a gentleMACs dissociator (Miltenyi Biotec, Bergisch Gladbach, Germany) with a M-tube. Brain samples were centrifuged at 5000 rcf for 5 min at 4 °C and the supernatant was collected for enzyme-linked immunosorbent assay. The experimental groups included normal, S/R, CMB + , and OMB + groups (each *N* = 5). The expression of endothelial nitric oxide synthase (eNOS; Cusabio, Wuhan, China), brain derived neurotrophic factor (BDNF; Cusabio), and NF-κB (ab176648, Abcam) was measured using commercial kits and protocols. Protein expression was detected by the optical density at 450 nm using a plate reader system (Tecan Infinite M200, Tecan Trading AG, Männedorf, Switzerland). The final protein expression was presented as the ratio relative to the normal cerebral hemisphere.

### Quantitative polymerase chain reaction

At 3 days after OMB treatment, the procedure of homogeneous brain sample collection for quantitative polymerase chain reaction was as described above. The experimental groups included normal, S/R, CMB + , and OMB + groups (each *N* = 5–9). Total mRNA was isolated from brain sample using Tri-reagent (TRIzol™ Reagent, Thermo Fisher Scientific, Waltham, USA). Primers for hypoxia-inducible factor 1-alpha (HIF-1α), B-cell lymphoma 2 (BCL2), interleukin 1 beta (IL-1β), interleukin 10 (IL-10), and matrix metallopeptidase 9 (MMP-9) were synthesized by Genomics BioSci and Tech Ltd. (New Taipei City, Taiwan) for SYBR green-based analysis. The final mRNA levels were normalized to β-actin expression and presented as ratios relative to normal brain. Details of the primers are provided in the Supplementary data (Table S1).

### Statistical analysis

Data were presented as the mean ± standard deviation from at least 3 independent experiments. Results were statistically analyzed by two-tailed, unpaired Student’s t-test for two-group comparisons. One-way ANOVA followed by Bonferroni's multiple comparisons test was used for comparisons of more than two groups. Statistically significant differences were considered for *p* < 0.05.

## Results

### In vitro OMB disruption by US sonication increased pO_2_ level

**Fig. 2:**
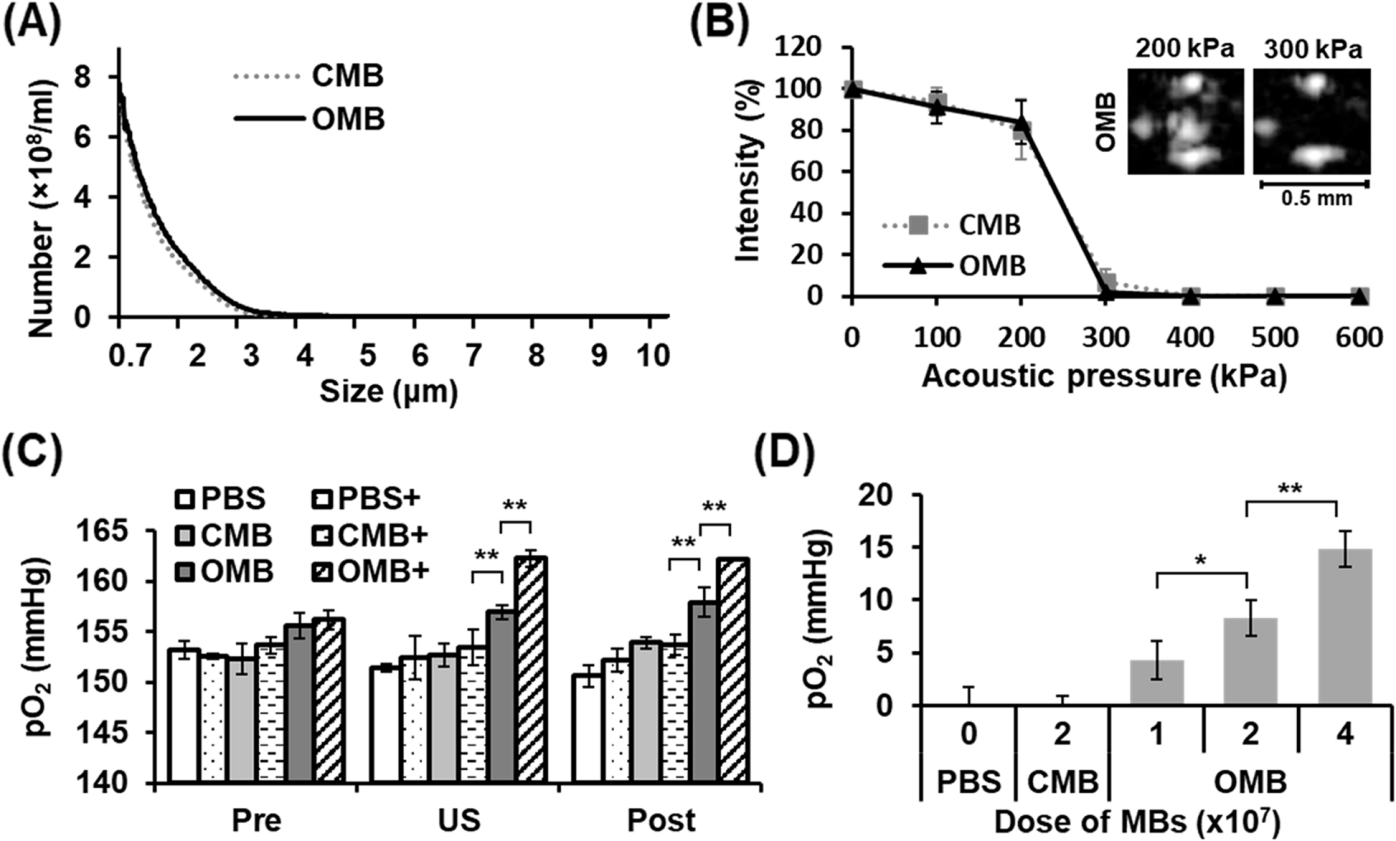
In vitro disruption of OMBs for O_2_ release. **A** The size distribution of CMBs and OMBs. **B** The destruction threshold of MBs under various acoustic pressures. US B-mode images of OMBs show contrast enhancement at an acoustic pressure of 200 kPa, but no enhancement at 300 kPa, defining the destruction threshold of OMB. **C** pO_2_ levels after OMB destruction by US with acoustic pressure of 300 kPa. The plus ( +) symbol presents US sonication. The concentration of CMBs and OMBs is 2 × 10^7^ MBs/mL. **D** Enhanced pO_2_ levels with increased doses of OMBs. Significant differences are indicated: *p < 0.05, **p < 0.01, by one-way ANOVA with Bonferroni's multiple comparisons test

The lipid-shell CMBs and OMBs had a mean diameter of 1.27 ± 0.61 and 1.33 ± 0.69 μm, respectively (Fig. 2A). The size distribution revealed that the diameter range was smaller than 4 μm in both the CMB and OMB groups. The in vitro US images showed only a slight decrease in contrast enhancement over time, demonstrating good stability of the CMBs and OMBs (Fig. S1). The contrast intensity of OMBs decreased from 24 ± 0.7 dB at 0 min to 23 ± 0.3 dB at 60 min. An acoustic pressure of 300 kPa was determined to be the destruction threshold for O_2_ therapy, and disrupted 93 ± 6.2% of CMBs and 98 ± 1.9% of OMBs (Fig. 2B). The intensity of contrast enhancement under US imaging decreased with an increase in acoustic pressures (Fig. S2). The results revealed no significant differences in size distribution, stability, and destruction threshold between CMBs and OMBs.

After US sonication, the pO_2_ levels increased by 0 ± 2, 0 ± 1, and 9 ± 2 mmHg in the PBS + , CMB + , and OMB + group, respectively (Fig. 2C). Final pO_2_ levels of the OMB group without and with US sonication were 158 ± 1 and 162 ± 0 mmHg, respectively, demonstrating local O_2_ release from OMBs triggered by US. The elevation of pO_2_ levels increased with increased OMB dose, further confirming the release of O_2_ from OMBs (Fig. 2D).

### The murine S/R model revealed non-complete recovery in blood flow, pO_2_, and brain infarct after thrombolysis

**Fig. 3:**
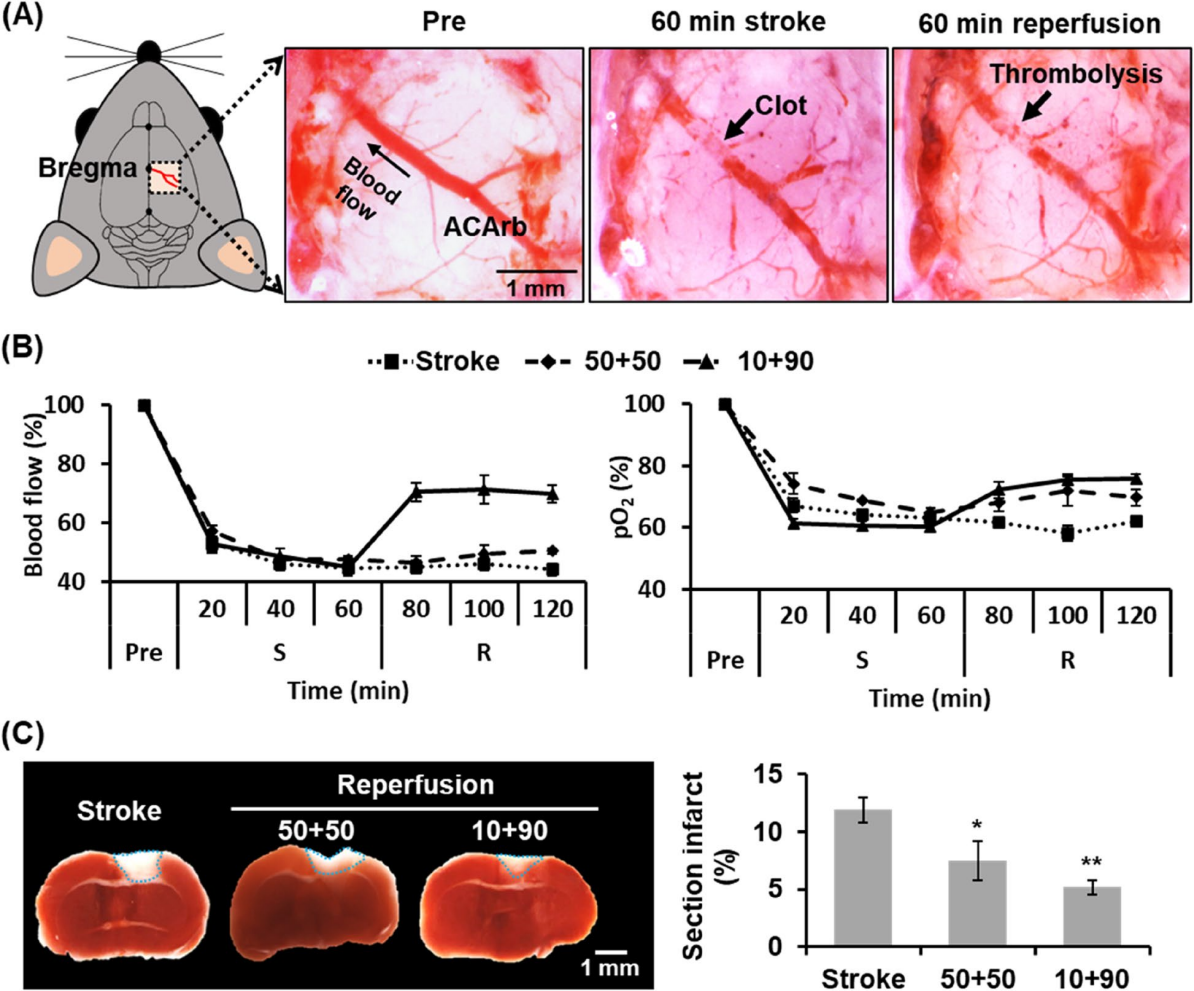
Assessment of murine S/R model. Methods of tPA administration included 50 μL bolus injection twice with an interval of 10 min (50 + 50) or 10 μL bolus injection and 90 μL continuous injection over 20 min (10 + 90). **A** Images from the dissecting microscope show normal vessel condition (pre), clot formation (60 min stroke), and thrombolysis (60 min reperfusion) in the 10 + 90 group. **B** Percentage of blood flow and pO_2_ during pre, stroke (S), and reperfusion (R). **C** Staining with 2% TTC shows white areas of brain infarction. The blue dotted outline marks the infarct areas. The quantified results present the percentage of the section with infarction. *N* = 3–5/group. Significant differences are indicated: *p < 0.05, **p < 0.01, by one-way ANOVA with Bonferroni's multiple comparisons test

During establishment of the murine S/R model, the dissecting microscopic images revealed clot formation at ACArb at 60 min after stroke induction and thrombolysis after 60 min of reperfusion (Fig. 3A). The optimal procedure for establishment of the murine S/R model was separated into stroke and reperfusion components. For the stroke component, photodynamic thrombosis with 20 mg/kg RB and 20 min laser exposure (20 + 20) significantly decreased the percentage of blood flow to 45 ± 3% and pO_2_ to 60 ± 1% at 60 min to establish the ischemic stroke model (Fig. S3A). TTC staining identified the brain infarcts as white regions at the right cerebral primary and secondary motor areas (Fig. S3B). The percentage of the infarct area was 0.0 ± 0.0, 5.1 ± 1.0, 5.4 ± 2.8, 8.0 ± 0.7, and 11.9 ± 1.1% in the normal, 10 + 20, 20 + 10, 20 + 15, and 20 + 20 groups, respectively.

In the reperfusion component, the 50 + 50 group with two bolus injections of tPA showed non-significant recovery in blood flow and pO_2_ (Fig. 3B). In contrast, blood flow and pO_2_ increased to 70 ± 3% and 76 ± 2% respectively in the 10 + 90 group, indicating successful thrombolysis for reperfusion after continuous injection of tPA. TTC staining showed a decrease in infarct size after reperfusion (Fig. 3C), with percentage infarct area of 11.9 ± 1.1, 7.5 ± 1.7, and 5.2 ± 0.6% in the stroke, 50 + 50, and 10 + 90 groups, respectively. Thus, in the present study the optimal procedure for generation of the murine S/R model was induction of stroke by photodynamic thrombosis with 20 mg/kg RB and 20 min laser exposure, followed by reperfusion by thrombolysis with 10 μL bolus injection and 90 μL continuous injection of tPA.

### OMB treatment promoted BBB opening without hemorrhage by regulating the stable cavitation of OMBs

**Fig. 4:**
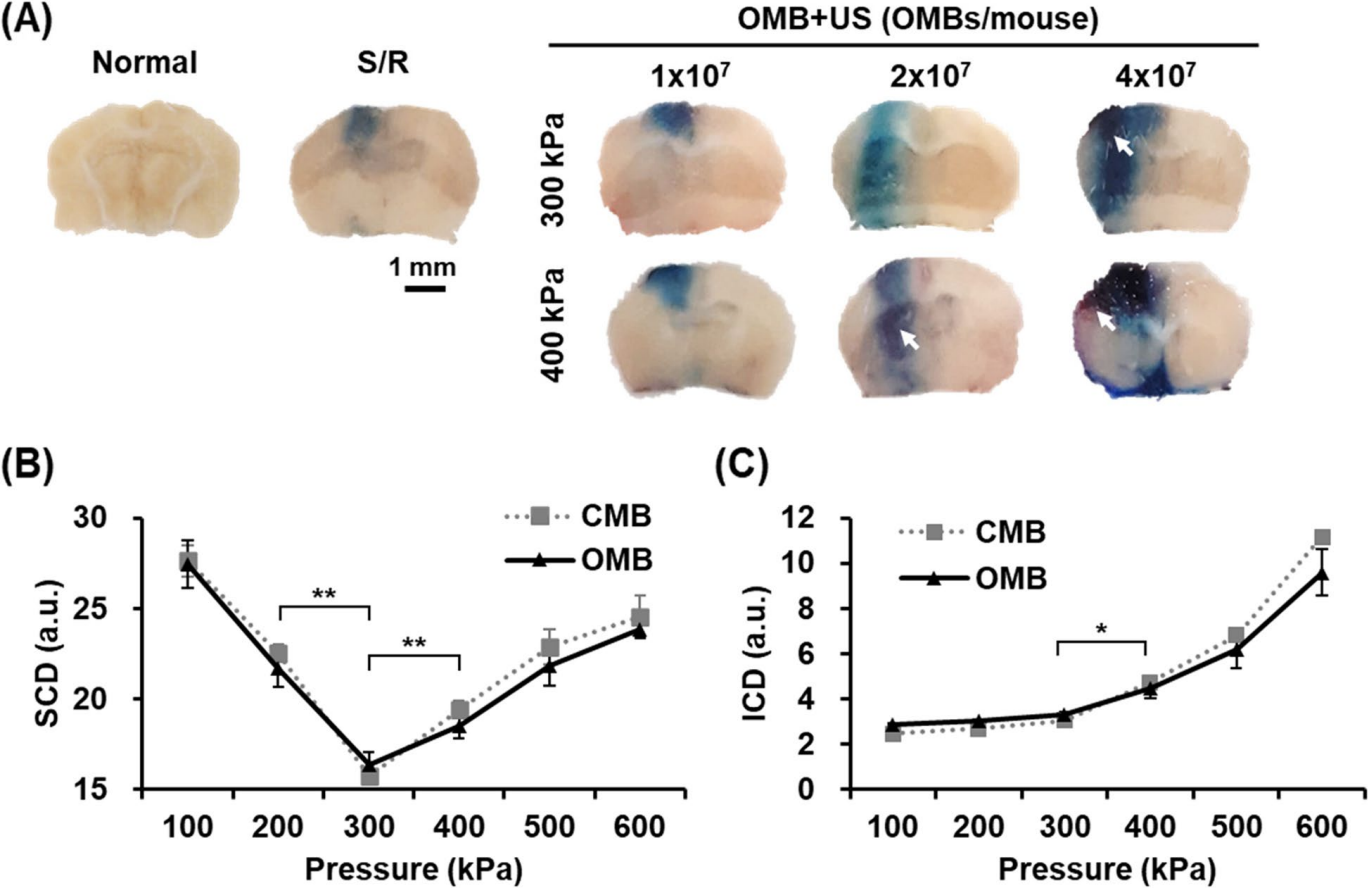
Biosafety of BBB opening induced by OMB cavitation. **A** The leakage of Evans blue dye within the murine S/R brains reveals the regions of BBB opening. The white arrows indicate brain hemorrhage. **B**, **C** In vitro detection of (**B**) SCD and (**C**) ICD of OMBs under different acoustic pressures. *N* = 3/group. Significant differences are indicated: *p < 0.05, **p < 0.01, by one-way ANOVA with Bonferroni's multiple comparisons test

In the S/R brain, leakage of Evans blue within the brain tissue indicated damage of the BBB due to the S/R injury (Fig. 4A). The degree of BBB opening was visibly enhanced after OMB cavitation; the blue areas within the S/R brain intensified after a dose of 1 × 10^7^ OMBs/mouse with acoustic pressures of 300 and 400 kPa. When the OMB dose was increased to 2 × 10^7^ OMBs/mouse, the areas of BBB opening were enlarged under an acoustic pressure of 300 kPa, but hemorrhage was induced at 400 kPa. Serious brain hemorrhage was observed in the groups treated with a dose of 4 × 10^7^ OMBs/mouse under acoustic pressures of both 300 and 400 kPa.

To evaluate the correlation between brain hemorrhage and OMB cavitation, the in vitro characteristics of OMB cavitation (2 × 10^7^ MBs/mL) under various acoustic pressures were examined. The stable cavitation of MBs produced at the acoustic pressures of 200 and 300 kPa due to the significant reduction of SCD, and then decreased at acoustic pressures of 400 kPa or higher (Fig. 4B). With conversion of OMB cavitation from stable to inertial cavitation, the ICD started to increase significantly at an acoustic pressure of 400 kPa (Fig. 4C). Thus, the optimal protocol of OMB treatment in the murine S/R model was defined as a treatment dose of 2 × 10^7^ OMBs/mouse with acoustic pressure of 300 kPa, which disrupted OMBs to release O_2_ and produced stable cavitation to promote BBB opening without brain hemorrhage.

### Blood flow and pO_2_ were recovered to reduce the infarct size after OMB treatment in the murine S/R model

**Fig. 5:**
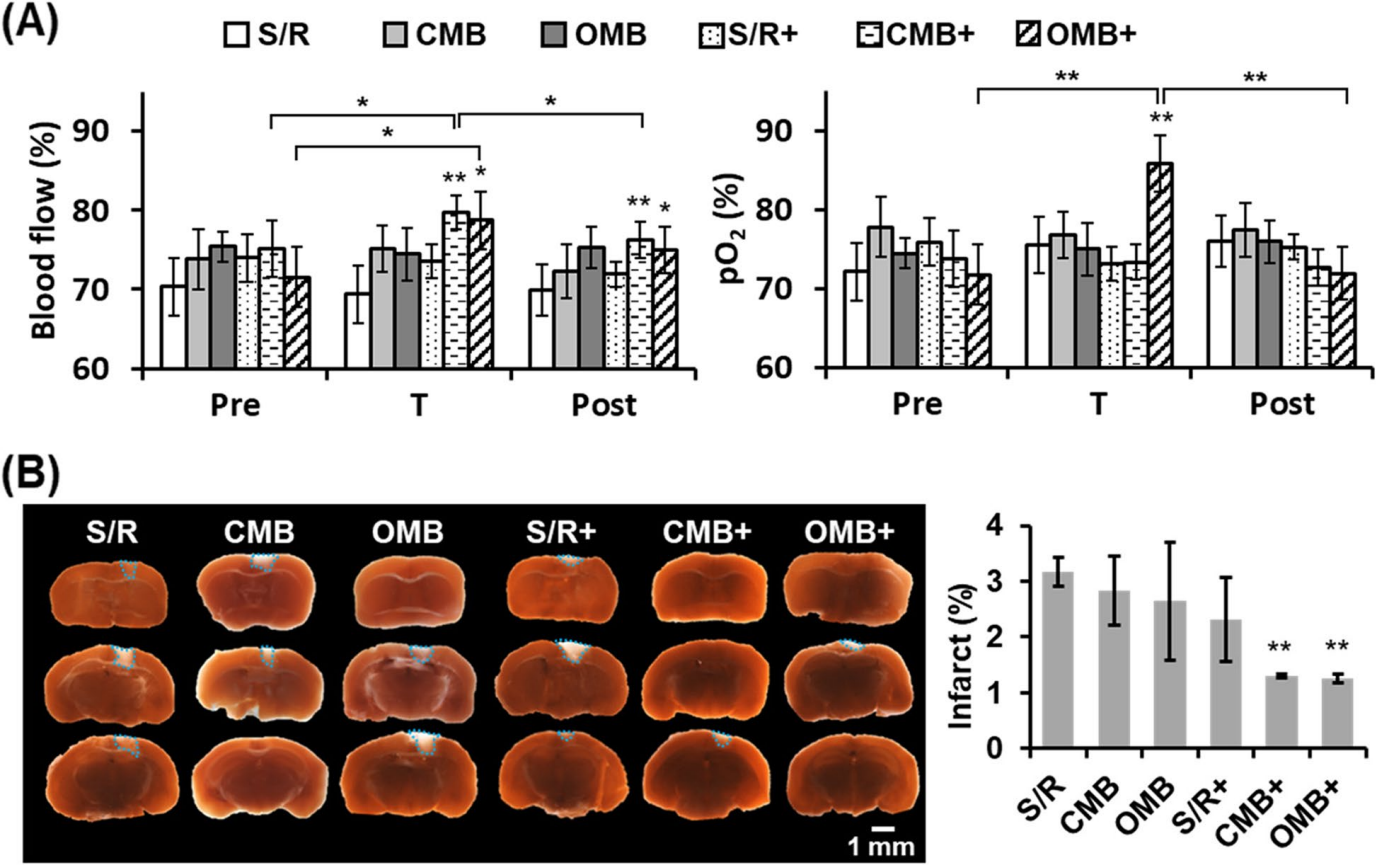
The in vivo effects of sonoperfusion and local O_2_ therapy by OMB treatment in murine S/R model. **A** The percentage of blood flow and pO_2_ at the periods of pre, during, and post OMB treatment (termed pre, T, and post, respectively). **B** Assessment of infarct size in different brain tissue sections. Staining with 2% TTC shows brain infarction as white areas. The blue dotted outline marks the infarct areas. The quantified results present the percentage of infarct area within the whole brain. The plus ( +) symbol represents US sonication. *N* = 5/group. Significant differences are indicated: *p < 0.05, **p < 0.01, by one-way ANOVA with Bonferroni's multiple comparisons test

To prevent S/R injury, S/R mice were subjected to OMB treatment at 20 min of reperfusion. The mean percentages of blood flow and pO_2_ were not significantly different between the pre, T, and post periods in the S/R, CMB, OMB, and S/R + groups (Fig. 5A). In the CMB + group, the blood flow was 75 ± 4%, 80 ± 2%, and 76 ± 2% at the pre, T, and post periods, respectively. A transient but significant enhancement of blood flow was induced by CMB cavitation but there was no significant difference in the pO_2_ levels. After OMB treatment (OMB +), the mean percentages of blood flow and pO_2_ were significantly increased by 7 ± 5% and 14 ± 4% at the T period relative to the pre period. In comparison with the T period, the blood flow decreased from 79 ± 4% to 75%3% (*p* > 0.05) and the pO_2_ decreased from 86 ± 2% to 72 ± 4% (*p* < 0.05) at the post period.

The white areas indicating infarct in brain sections stained by TTC showed a reduction in infarct size in the CMB + and OMB + groups (Fig. 5B). The percentages of infarct area within the whole brain were 3.2 ± 0.3%, 2.8 ± 0.6%, 2.6 ± 1.1%, 2.3 ± 0.8%, 1.3 ± 0.0%, and 1.3 ± 0.1% in the S/R, CMB, OMB, S/R + , CMB + , and OMB + , respectively. A significant reduction in infarct size in whole brain demonstrated the efficacy of S/R injury prevention by both CMB and OMB treatment.

### Long-term tracing of animal behaviors and brain infarct size revealed rapid recovery of brain function after OMB treatment

**Fig. 6:**
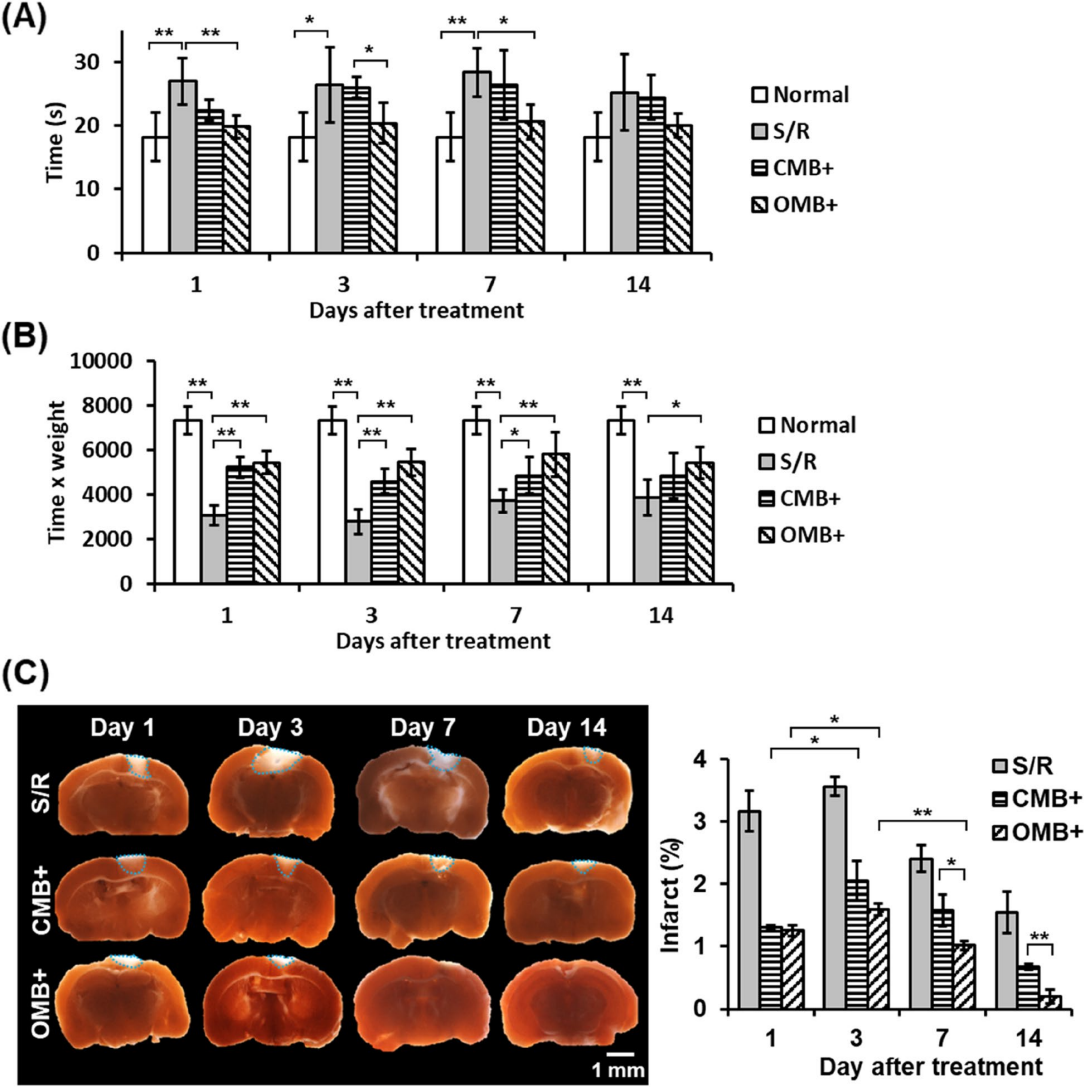
Long-term tracking of animal behaviors and size of brain infarct in murine S/R model. **A**, **B** Quantitative results of **A** beam walking test and **B** hanging wire test. *N* = 4–5/group. **C** Assessment of infarct size within whole brains. Staining with 2% TTC shows brain infarction as white areas. The blue dotted outline marks the infarct areas. The quantified results present the percentage of infarct area within the whole brain. *N* = 3/group. The plus ( +) symbol represents US sonication. Significant differences are indicated: *p < 0.05, **p < 0.01, by one-way ANOVA with Bonferroni's multiple comparisons test

The recovery of brain function after OMB treatment was evaluated through long-term tracking of mice behaviors and TTC staining. The results of the beam walking test and hanging wire test showed a significant difference between the normal and S/R groups that demonstrated damage to brain function after S/R (Fig. 6A and B). In the OMB + group, the time of the beam walking test decreased after day 1 and showed no significant difference from the normal group at day 7. There was no significant difference among all groups at day 14, with values of 18 ± 4, 25 ± 6, 24 ± 3, 20 ± 2 s in the normal, S/R, CMB + , and OMB + groups, respectively. The results of the hanging wire test at 14 days after treatment showed a significant increase in the CMB + and OMB + groups but the values did not recover to the normal level.

TTC staining showed the maximum brain infarct size at day 3 and a subsequent reduction in size with time in each group (Fig. 6C). The brain infarct areas were 3.6 ± 0.2%, 2.1 ± 0.3%, and 1.6 ± 0.1% at day 3 in the S/R, CMB + , and OMB + group, respectively, compared with 1.6 ± 0.3%, 0.7 ± 0.0%, and 0.2 ± 0.1% at day 14. In comparison with CMB treatment, OMB treatment resulted in a faster recovery of animal behaviors and brain infarct sizes.

### Reoxygenation and sonoperfusion protected neurons through inhibition of the activation of inflammatory and apoptotic factors

**Fig. 7:**
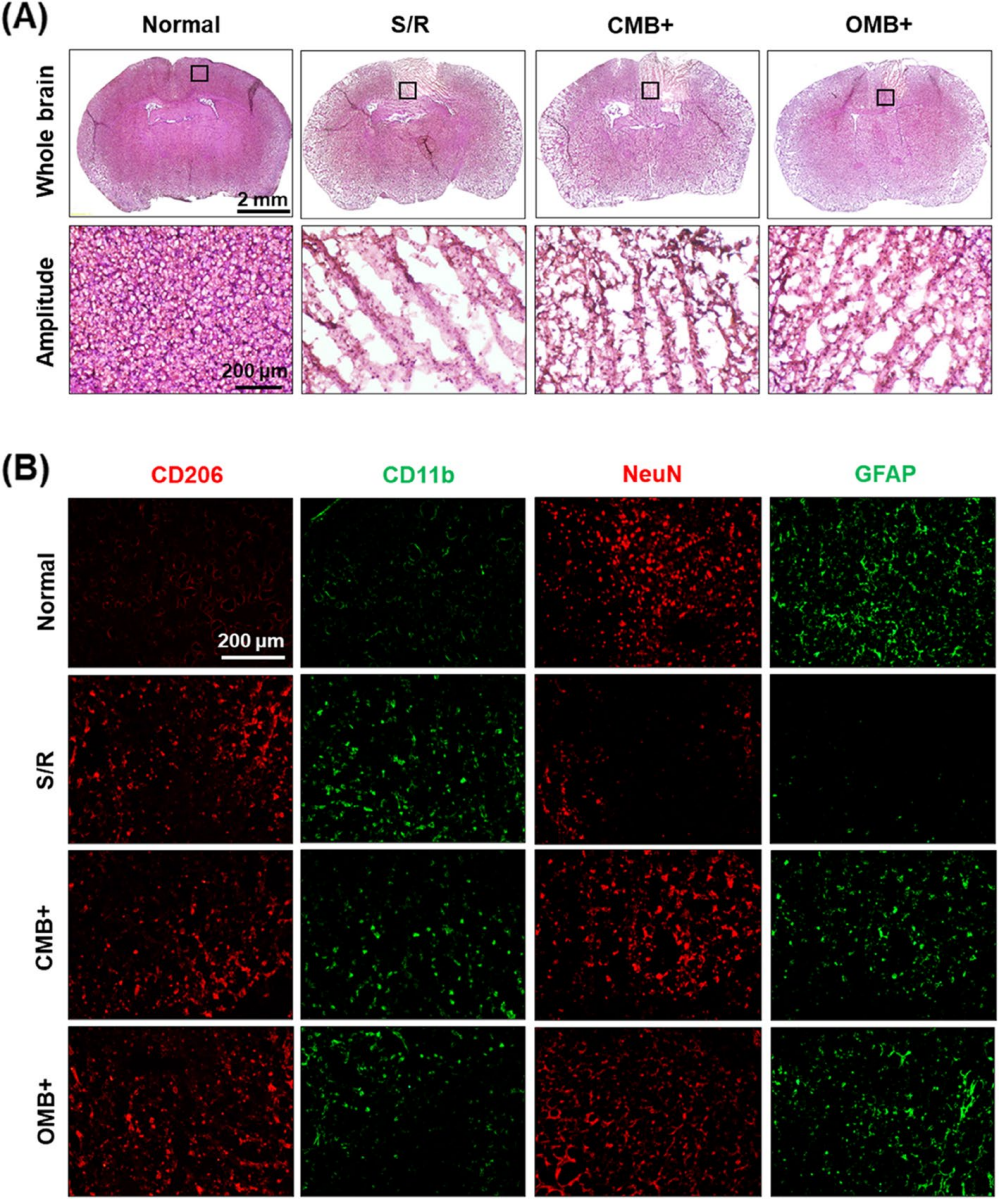
Histologic qualitative assessment of murine S/R model after OMB treatment for 3 days. **A** H&E images of whole brains. The black squares indicate the locations of the amplitude images. **B** Immunohistochemical staining of CD206, CD11b, NeuN, and GFAP to evaluate the distribution of anti-inflammatory M2 microglia, pro-inflammatory M1 microglia, mature neurons, and astrocytes, respectively, within the brain infarct areas. The plus ( +) symbol represents US sonication

After 3 days of OMB treatment, histological staining, protein expression, and mRNA levels in the brain were evaluated. The histological images revealed sparse tissue in the S/R group that became denser in the CMB + and OMB + groups (Fig. 7A). Immunohistochemical staining of CD206 showed numerous anti-inflammatory M2 microglia in the S/R, CMB + , and OMB + groups (Fig. 7B). In comparison to the S/R group, the CMB + and OMB + groups showed a decrease in CD11b labeling demonstrating a reduction in pro-inflammatory M1 microglia distribution. The absence of mature neurons and astrocyte distribution in the S/R group reflected the abnormal animal behaviors caused by the dysfunctional brain.

**Fig. 8:**
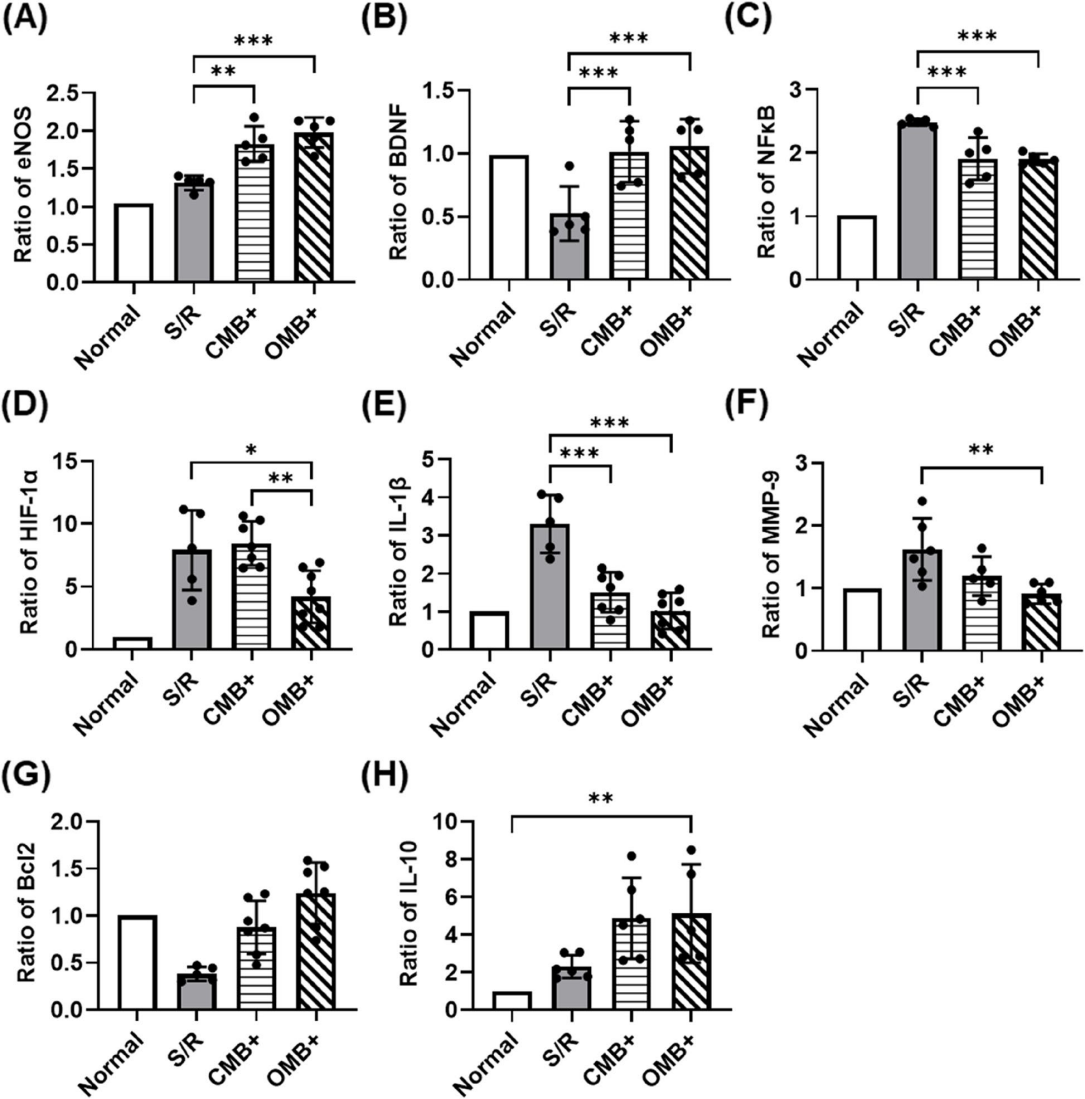
Ratios of protein and mRNA expression within the S/R cerebral hemispheres relative to the normal cerebral hemispheres after OMB treatment for 3 days. **A**-**C** The protein expression levels of eNOS, BDNF, and NF-κB reveal the activation of vasodilation, neuron growth, and inflammatory response, respectively. *N* = 5/group. **D**-**H** The mRNA expression levels of HIF-1α, IL-1β, MMP-9, BCL2, and IL-10 indicate hypoxia, pro-inflammatory M1 microglia, BBB disruption, anti-apoptosis, and anti-inflammatory M2 microglia, respectively.* N* = 5–9/group. The plus ( +) symbol represents US sonication. Significant differences are indicated: *p < 0.05, **p < 0.01, ***p < 0.001, one-way ANOVA with Bonferroni's multiple comparisons test

Expression of proteins and mRNA in the S/R cerebral hemispheres was also detected to evaluate the mechanisms of local O_2_ therapy and sonoperfusion induced by OMB treatment. Consistent with the mechanical effect of MB cavitation on the vessel wall, the expression of eNOS for vasodilation was significantly enhanced in the CMB + and OMB + groups relative to the S/R group (Fig. 8A). The expression of BDNF was 1.0 ± 0.0, 0.5 ± 0.2, 1.0 ± 0.2, and 1.1 ± 0.2 in the normal, S/R, CMB + , and OMB + groups, respectively, indicating the recovery of BDNF expression for promotion of neuron growth after treatment (Fig. 8B). The significant decrease in expression of NF-κB in the CMB + and OMB + groups indicated inhibition of the inflammatory response after MB cavitation (Fig. 8C). The mRNA ratios showed significant decreases in HIF-1α, IL-1β, and MMP-9, representing reoxygenation, reduction of pro-inflammatory M1 microglia, and BBB recovery, respectively, after OMB treatment (Fig. 8D, E, and F). Moreover, the increase in Bcl2 and IL-10 mRNA levels revealed anti-apoptosis activity and an increase in anti-inflammatory M2 microglia differentiation in the OMB + group (Fig. 8G and H). Changes in mRNA levels in the CMB group were not significantly different from the S/R group except for the significant reduction of IL-1β. Comparisons between the normal, S/R, and contralateral cerebral hemispheres are shown in the Supplementary data (Figure S5).

## Discussion

US-stimulated MB cavitation has been widely applied in the treatment of various diseases because of its combined local drug delivery and acousto-mechanical vascular effects. The present study used OMB treatment to prevent murine S/R injury after thrombolysis. Sonoperfusion induced during OMB treatment might be responsible for the 9.4 ± 5.1% enhancement in blood flow observed relative to the S/R group. The improvement of blood perfusion signals at the peripheral microcirculation of ACArb after OMB treatment was visualized in the laser speckle images (Fig. S4). Since MB cavitation can activate release of the vasodilator nitric oxide (NO), sonoperfusion might promote effective reperfusion in the microcirculation to protect neurons [18, 25]. In addition, the local release of O_2_ during OMB treatment promoted a 10.3 ± 3.1% increase in pO_2_ level relative to the S/R group, allowing reoxygenation of the hypoxic brain. This enhanced reoxygenation could reduce the pathological effects of hypoxia and ischemia, effectively reducing the infarct size of the ischemic brain. In our study, the infarct size was not significantly different between the CMB + and OMB + groups at days 1 to 3, but significantly decreased in the OMB + group relative to the CMB + group at days 7 to 14. This reduction of infarct size demonstrated that the reoxygenation effect of OMB treatment might provide long-term recovery of the ischemic penumbra to protect brain function related to limb coordination [26]. The mRNA expression levels at different time points would be further evaluated to elucidate the biomechanism of CMB and OMB treatments in our future work.

Based on the mechanism of O_2_ therapy, hyperbaric O_2_ therapy (HBOT) and normobaric O_2_ therapy (NBOT) have been applied the prevention of S/R injury in clinical applications or trials [26–28]. The production of reactive oxygen species (ROS) at non-lethal levels during HBOT activates the expression of antioxidant enzymes to balance the oxidative stress [29, 30]. The increase of pO_2_ levels in the cerebral ischemic penumbra could reduce the infarct formation and active neuroprotective effects [26, 31]. However, the whole-body effect of HBOT and NBOT should be concerned the duration and repetition times to avoid excessive production of ROS and central nervous system O_2_ toxicity [32, 33]. Therefore, the local O_2_ release of OMB treatment might represent a promising strategy to achieve safer and more precise O_2_ therapy to prevent the systemic side effects. To date, OMB treatment has been used in tumor therapy to reoxygenate hypoxic tumors and improve the efficacy of radiotherapy and chemotherapy [21, 23, 34, 35]. The increase in ROS production via OMB cavitation has also been exploited to improve the efficacy of sonodynamic therapy [19, 36]. However, when applying OMB treatment in normal organs or tissues, the production of cytotoxic ROS during OMB cavitation should be considered to avoid further injury. Importantly, ROS do not only induce cytotoxicity, but also regulate cellular metabolism for cell proliferation, differentiation, migration, and repair [37]. Intracellular ROS levels in normal cells should therefore be maintained at a homeostatic level through effective antioxidant production [38]. In the current study, the prevention and repair of S/R injury by OMB treatment was demonstrated by the recovery of brain morphology and function. Changes in ROS levels in the murine S/R model should be measured to investigate the level of oxidative stress after OMB treatment.

**Fig. 9:**
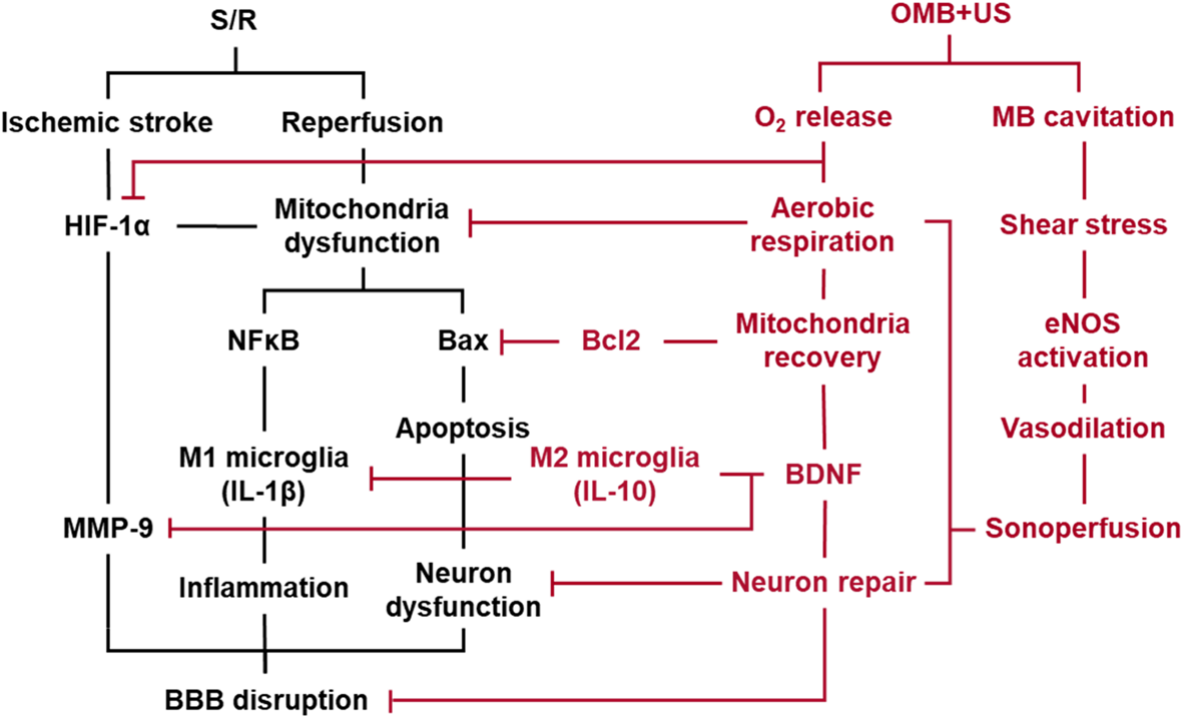
The biomechanism of OMB treatment for S/R injury prevention can be broadly separated into local O_2_ release and MB cavitation. The enhancement of endothelial shear stress activates eNOS for vasodilation, causing sonoperfusion. The effects of sonoperfusion and local O_2_ release promote cellular aerobic respiration, inhibiting HIF-1α expression, mitochondrial dysfunction, and subsequent apoptosis. The release of BDNF protects neurons, reducing inflammatory responses and BBB disruption to prevent S/R injury and protect neuron function

The biomechanisms of OMB treatment included the activation of eNOS for vasodilation, BDNF for neuron repair, IL-10 for anti-inflammatory M2 microglia differentiation, and Bcl2 for anti-apoptosis activity (Fig. 9). Inhibition of HIF-1α, NF-κB, and IL-1β expression indicated a reduction of brain hypoxia and inflammatory responses by OMB treatment. The decrease in MMP-9 after OMB treatment would reduce disruption of the BBB induced by S/R injury. Moreover, the contralateral cerebral hemispheres in each group showed no significant difference in the expression of eNOS, IL-1β, MMP-9, and BCL2 relative to the normal cerebral hemispheres (Fig. S5). The expression of anti-inflammatory IL-10 in the contralateral cerebral hemispheres was enhanced in each group, possibly to balance the pro-inflammatory IL-1β in the S/R cerebral hemispheres. The regulation of inflammatory responses in the contralateral cerebral hemispheres might promote the expression of growth factors and immune modulators for neuron proliferation and survive, which might maintain the brain function and support the S/R cerebral hemispheres [39]. HIF-1α expression in the contralateral cerebral hemispheres was not significantly different from than that in the S/R cerebral hemispheres in the S/R and CMB + groups but was significantly higher for the OMB + group. Stabilization of HIF-1α in the contralateral cerebral hemispheres might help to decrease injury of the S/R cerebral hemispheres. Hypoxia-mimetic agents (e.g., HIF-prolyl hydroxylase inhibitor) have been reported to prevent S/R injury by neuroprotection, angiogenesis, and erythropoiesis through the stabilization of HIF-1α expression [40]. However, the efficacy of hypoxia-mimetic agents for preventing S/R injury is a controversial issue, and the timing and levels of HIF-1α expression during the treatment should be further investigated to avoid aggravation of injury [41]. Even though the expression of MMP-9 was reduced after OMB treatment, the induction of BBB opening by MB cavitation might enhance penetration of inflammatory factors into the injured brain tissue and impede recovery [42]. In normal brain, the expression of inflammatory factors was significantly increased at 6 h and 24 h after BBB opening induced by MB cavitation and returned to the baseline level after 72 h [43]. Thus, the optimal time point of OMB treatment, the duration of BBB opening, and the possibility of BBB recovery should be evaluated to avoid aggravation of S/R injury.

Sonothrombolysis has been applied to promote fibrinolytic therapy via MB cavitation [13]. The mechanical force induced by MB cavitation disrupts the thrombus, allowing tPA to penetrate into deep sites for thrombolysis [44]. The recovery of blood flow and vascular recanalization play a crucial role in preventing ischemia–reperfusion injury in thrombolysis. Corro et al. designed a NO donor-loaded MB to induce rat femoral vasodilation and clot degradation by sonothrombolysis for femoral artery recanalization [45]. The local release of NO from NO donor-loaded MBs enhanced the relaxation of rat aortic rings to achieve the NO-related vasodilation effect. In the present study, OMB treatment was performed after blood reperfusion for 20 min to prevent S/R injury. However, different time schedules for ischemia stroke, thrombolytic reperfusion, and OMB treatment should be further investigated to determine the optimal treatment procedure for preventing S/R injury. Figure S6A shows initial results for OMB treatment at the late stage of stroke (S), early stage of reperfusion (S-R), and post reperfusion (R). The percentage of infarct areas within whole brain at 24 h after treatment was 2.4 ± 0.7, 1.0 ± 0.4, and 1.6 ± 0.1 in the S, S-R, and R groups, respectively (Fig. S6B). The combination of tPA administration and OMB treatment in the S-R group promoted sonothrombolysis to significantly shrink the subsequent brain infarct area.

## Conclusion

This study demonstrated that US-stimulated OMB cavitation could prevent and repair S/R injury. The enhanced blood flow induced by sonoperfusion promoted blood reperfusion at the peripheral microcirculation to supply nutrients to neurons. Reoxygenation of the ischemic brain by local O_2_ therapy inhibited hypoxia-inducible inflammation and apoptosis to prevent subsequent injury. Recovery of the reversible ischemic penumbra reduced brain infarct size. Brain morphology and function in S/R mice were protected to maintain the ability of limb coordination due to the survival of neurons and astrocytes. This local and non-invasive OMB treatment might be a promising strategy for the development of a precise therapy to prevent ischemia–reperfusion injury in various organ systems.

## Supplementary Information

**Additional file 1.**

## Data Availability

The datasets used and/or analyzed during the current study are available from the corresponding author on reasonable request.

## Abbreviations

tPA: Tissue plasminogen activator
FDA: United States Food and Drug Administration
S/R: Ischemic stroke-reperfusion
BBB: Blood–brain barrier
US: Ultrasound
MBs: Microbubbles
MBs: Oxygen-loaded MBs
PRF: Pulse repetition frequency
pO_2_: Partial pressure of O_2_
SCD: Stable cavitation dose
ICD: Inertial cavitation dose
ACArb: Anterior cerebral artery remote branch
RB: Rose bengal
TTC: 2,3,5-triphenyltetrazolium chloride
H&E: Hematoxylin and eosin staining
eNOS: Endothelial nitric oxide synthase
BDNF: Brain derived neurotrophic factor
HIF-1α: Hypoxia-inducible factor 1-alpha
BCL2: B-cell lymphoma 2
IL-1β: Interleukin 1 beta
IL-10: Interleukin 10
MMP-9: Matrix metallopeptidase 9
NO: Nitric oxide
HBOT: Hyperbaric O_2_ therapy
NBOT: Normobaric O_2_ therapy
ROS: Reactive oxygen species
