# Supplementary material for: Oxygen-loaded microbubble-mediated sonoperfusion and oxygenation for neuroprotection after ischemic stroke reperfusion
Source: Biomater Res. 2023 Jul 6;27:65. doi: 10.1186/s40824-023-00400-y (PMC10324216; doi:10.1186/s40824-023-00400-y)
Supplement: Supplementary file 1 — Additional file 1. [file 40824_2023_400_MOESM1_ESM.docx]

**Oxygen-loaded microbubble-mediated sonoperfusion and oxygenation for neuroprotection after ischemic stroke reperfusion**

Yi-Ju Ho^1^, Hsiang-Lung Cheng^2^, Lun-De Liao^3^, Yu-Chun Lin^4, 5^, Hong-Chieh Tsai^6,7,*^, and Chih-Kuang Yeh^2,*^

**Supplemental Information**

Table S1

Figures S1-S6

**Table S1.** Primer sequences for qPCR.

**
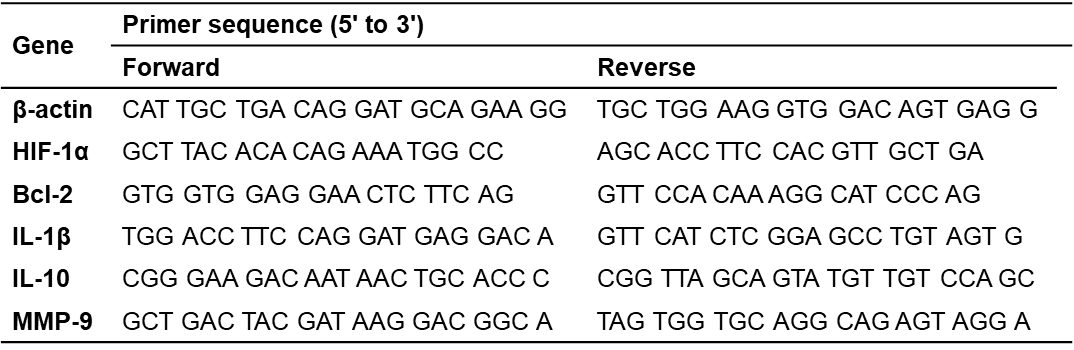
**


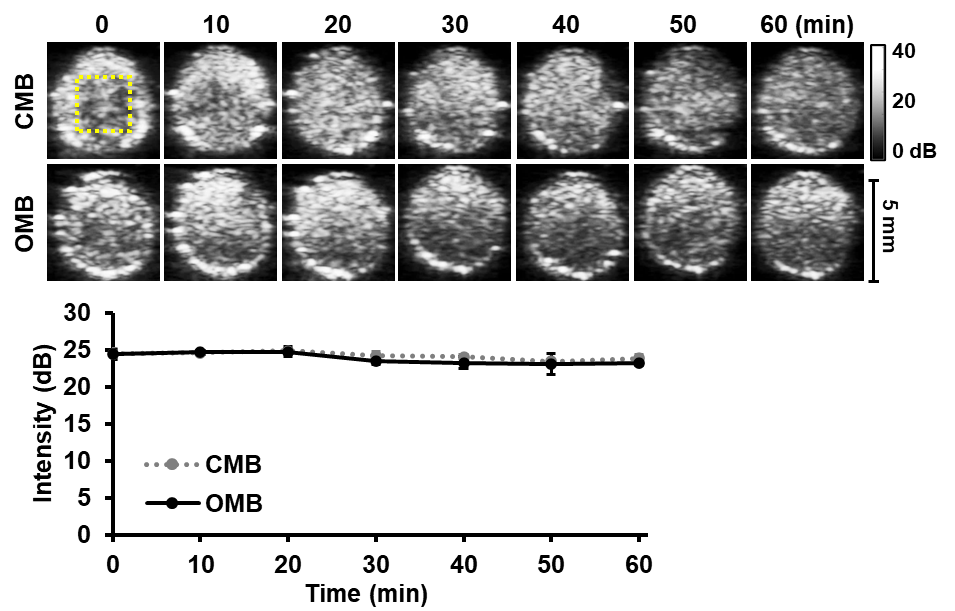


**Figure S1. *In vitro* stability of oxygen-loaded microbubbles (OMB) and C_3_F_8_-loaded microbubbles (CMB) under ultrasound B-mode imaging.** The MB emulsion (2x10^7^ MBs/mL) was added to a cylindrical hollow chamber of an agarose phantom. **(A)** The ultrasound B-mode images were recorded over time to analyze the change in image contrast enhancement. The series of images show a slight reduction in contrast enhancement over time in both the OMB and CMB groups. The yellow square indicates the region of interest (ROI; 2.5x2.5 mm^2^) for quantification of contrast intensity. **(B)** The contrast intensity of OMBs was 24±0.7 dB at 0 min and 23±0.3 dB at 60 min (*p*=0.042).


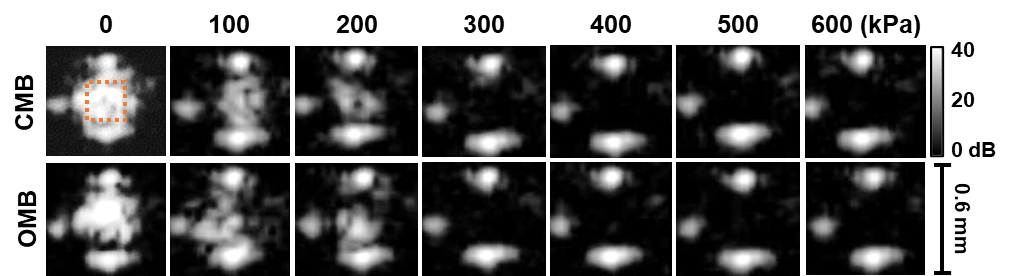


**Figure S2. The destruction threshold of oxygen-loaded microbubbles (OMB) and C_3_F_8_-loaded microbubbles (CMB) under different acoustic pressures.** The MB emulsion (2x10^7^ MBs/mL) was infused into a hollow pipe (Ø=0.58 mm) of an agarose phantom to mimic the vessel condition. The ultrasound B-mode images were recorded over time to analyze the reduction of image contrast enhancement when MBs are disrupted by a 1-MHz focused ultrasound transducer. The destruction threshold of OMBs and CMBs is defined when the ultrasound images show no contrast enhancement under focused ultrasound stimulation with acoustic pressure of 300 kPa. The yellow square indicates the region of interest (ROI; 0.25x0.25 mm^2^) for quantification of contrast intensity.


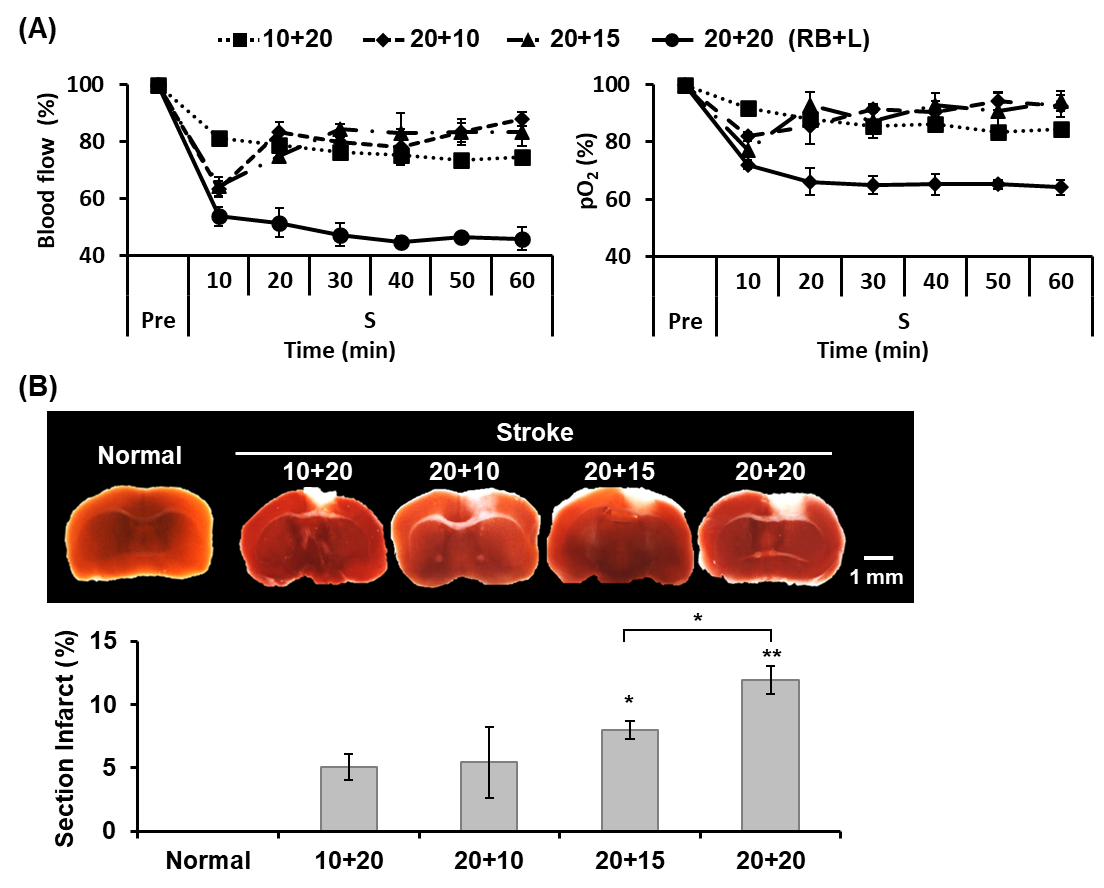


**Figure S3. Establishment of murine ischemic-stroke model via photodynamic thrombosis.** The photodynamic thrombosis is formed at the murine anterior cerebral artery remote branch (ACArb). Mice were retro-orbitally injected with 50 μL of photosensitizer rose bengal at doses of 10 and 20 mg/k and then exposed to a 0.5 mW laser with wavelength of 532 nm for 10, 15, or 20 min at the ACArb. **(A)** The group names are based on the doses of rose bengal plus the exposure time of laser (RB+L). The blood flow was 74±0%, 88±3%, 83±6%, and 46±5% and the pO_2_ level was 84±1%, 93±5%, 94±4%, and 64±3% in the 10+20, 20+10, 20+15, and 20+20 groups, respectively. In the 20+20 group, the blood flow and pO_2_ levels continuously decreased and were maintained at significantly low levels at 60 min indicating stable clot formation. **(B)** The brain infarct area was stained white by 2% 2,3,5-triphenyltetrazolium chloride (TTC). The percentage of section with infarct was 0.0+0.0%, 5.1+1.0%, 5.4+2.8%, 8.0+0.7%, and 11.9+1.1% in the 10+20, 20+10, 20+15, and 20+20 groups, respectively. *N*=3/group. Significant differences are indicated: **p*<0.05, ***p*<0.01, by one-way ANOVA with Bonferroni's multiple comparisons test.


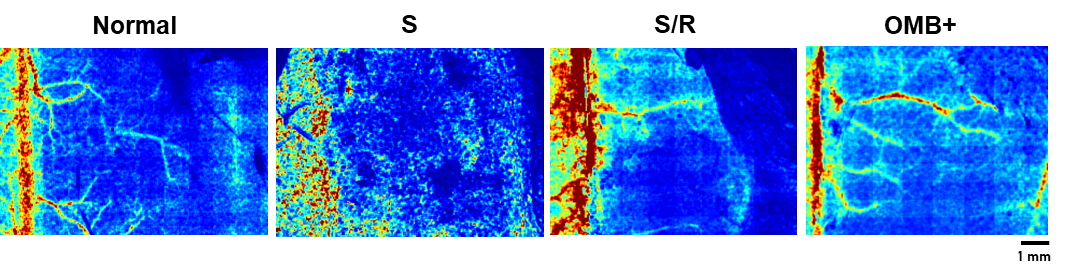


**Figure S4. OMB treatment-induced sonoperfusion increased cerebral microcirculation.** Blood perfusion images at the murine anterior cerebral artery remote branch (ACArb) were collected using a laser speckle imaging system (RFLSI III, RWD Life Sciences, Shenzhen, China). The normal image reveals weak signals at ACArb, then shows no signals at stroke (S) period to indicate the formation of ischemic stroke. After thrombolysis, the blood perfusion signals at ACArb are increased in the S/R image. The improvement of blood perfusion signals at the peripheral microcirculation of ACArb is visualized after OMB treatment (OMB+). The mice are different in each image.


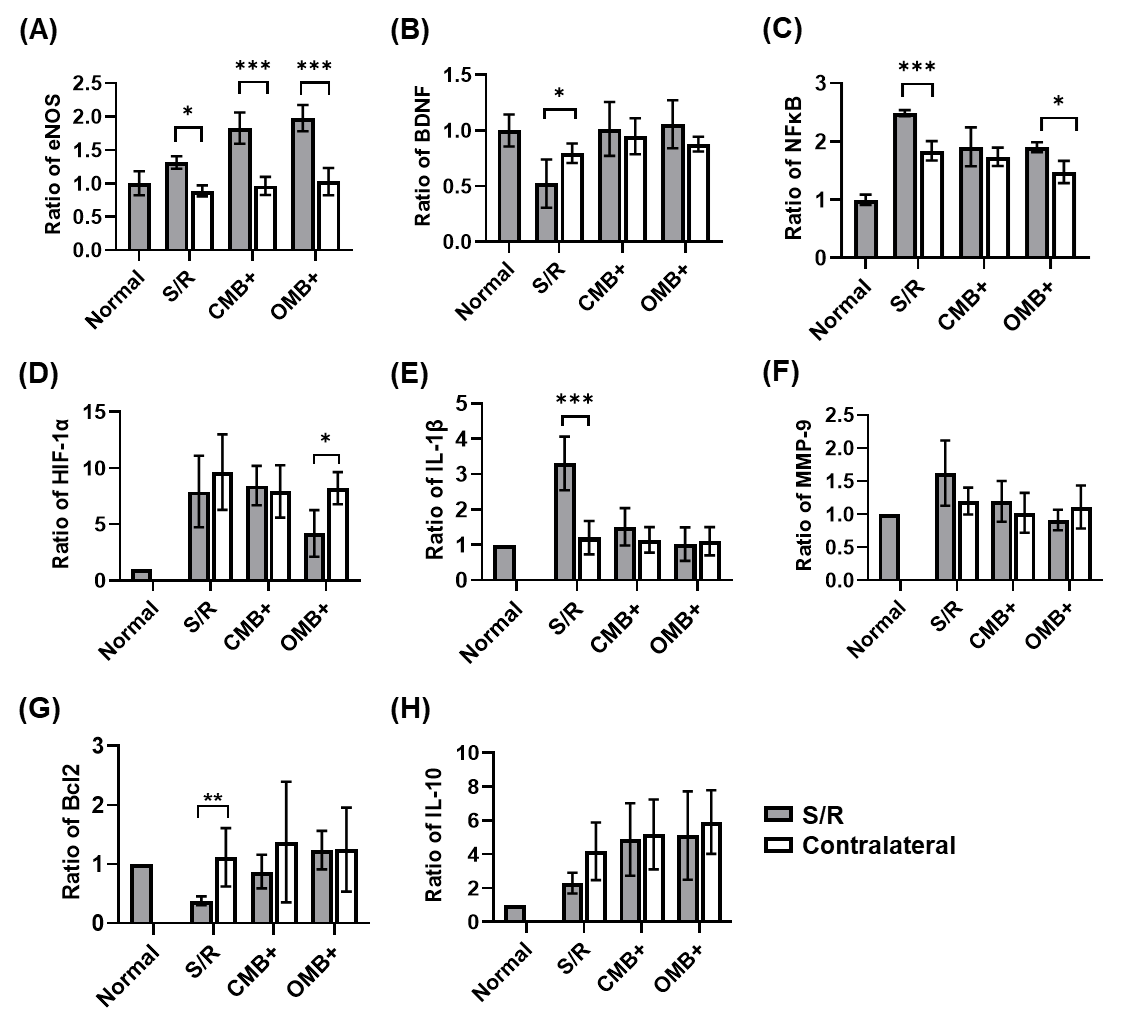


**Figure S5. Comparison of protein and mRNA expression between the S/R and contralateral** **cerebral hemispheres.** After OMB treatment for 3 days, the mice were sacrificed with perfusion and the mouse brain was divided into two cerebral hemispheres, the S/R cerebral hemisphere and the contralateral cerebral hemisphere. **(A-C)** Protein expression levels of endothelial nitric oxide synthase (eNOS), brain derived neurotrophic factor (BDNF), and NF-κB. Compared with the normal brain, the contralateral cerebral hemispheres showed no significant difference in eNOS expression and overexpression of NF-κB for each group. Only the S/R group showed a significant decrease in BDNF expression relative to the normal group. **(D-H)** The mRNA expression of hypoxia-inducible factor 1-alpha (HIF-1α), interleukin 1 beta (IL-1β), matrix metallopeptidase 9 (MMP-9), B-cell lymphoma 2 (BCL2), and interleukin 10 (IL-10). The results showed no significant difference between the normal cerebral hemispheres and contralateral cerebral hemispheres in the expression of IL-1β, MMP-9, and BCL2 in each group. Increases in HIF-1α and IL-10 expression relative to the normal cerebral hemisphere were observed in the S/R, CMB+, and OMB+ groups. *N*=5-9/group. The plus (+) symbol represents US sonication. Significant differences are indicated: **p*<0.05, ***p*<0.01, ****p*<0.001 by one-way ANOVA with Bonferroni's multiple comparisons test.


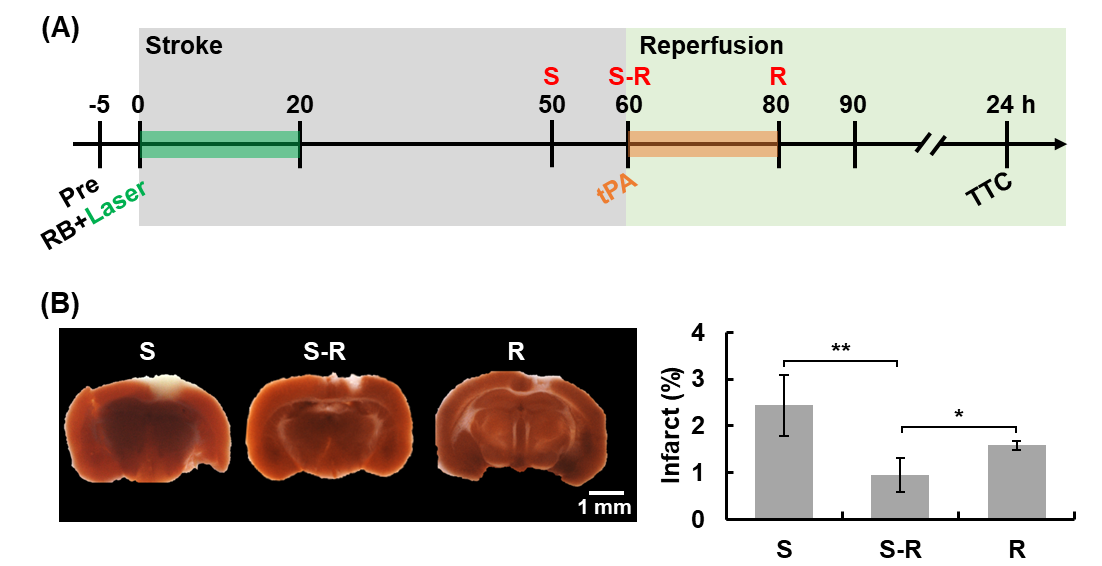


**Figure S6. (A)** Flow chart of OMB treatment at different time schedules. The treatment time points are labeled at 50 min indicating the late stage stroke (S), 60 min indicating the early-stage reperfusion (S-R), and 80 min indicating the post reperfusion (R). OMB treatment at the end of the stroke period was implemented before reperfusion for preprocessing. At the S-R time point, OMB emulsion was injected immediately after bolus injection of tPA to combine thrombolysis, oxygen therapy, and sonoperfusion on the same temporal schedule. **(B)** The brain infarct areas are stained white by TTC . The percentage of infarct areas within the whole brain shows the minimum infarct size in the S-R group. *N*=3/group. Significant differences are indicated: **p*<0.05, ***p*<0.01, by one-way ANOVA with Bonferroni's multiple comparisons test.
